# Biodegradability of Dental Care Antimicrobial Agents Chlorhexidine and Octenidine by Ligninolytic Fungi

**DOI:** 10.3390/molecules25020400

**Published:** 2020-01-18

**Authors:** Lucie Linhartová, Klára Michalíková, Kamila Šrédlová, Tomáš Cajthaml

**Affiliations:** 1 Laboratory of Environmental Biotechnology, Institute of Microbiology of the Czech Academy of Sciences, Vídeňská 1083, CZ-14220 Prague 4, Czech Republic; lucie.linhartova@biomed.cas.cz (L.L.); klara.michalikova@biomed.cas.cz (K.M.); kamila.sredlova@biomed.cas.cz (K.Š.); 2 Institute for Environmental Studies, Faculty of Science, Charles University, Benátská 2, CZ-12801 Prague 2, Czech Republic

**Keywords:** chlorhexidine, dental hygiene, laccase, manganese-dependent peroxidase, octenidine, ligninolytic fungi, personal care products, quaternary ammonium compounds, recalcitrant pollutant

## Abstract

Chlorhexidine (CHX) and octenidine (OCT), antimicrobial compounds used in oral care products (toothpastes and mouthwashes), were recently revealed to interfere with human sex hormone receptor pathways. Experiments employing model organisms—white-rot fungi *Irpex lacteus* and *Pleurotus ostreatus*—were carried out in order to investigate the biodegradability of these endocrine-disrupting compounds and the capability of the fungi and their extracellular enzyme apparatuses to biodegrade CHX and OCT. Up to 70% ± 6% of CHX was eliminated in comparison with a heat-killed control after 21 days of in vivo incubation. An additional in vitro experiment confirmed manganese-dependent peroxidase and laccase are partially responsible for the removal of CHX. Up to 48% ± 7% of OCT was removed in the same in vivo experiment, but the strong sorption of OCT on fungal biomass prevented a clear evaluation of the involvement of the fungi or extracellular enzymes. On the other hand, metabolites indicating the enzymatic transformation of both CHX and OCT were detected and their chemical structures were proposed by means of liquid chromatography–mass spectrometry. Complete biodegradation by the ligninolytic fungi was not achieved for any of the studied analytes, which emphasizes their recalcitrant character with low possibility to be removed from the environment.

## 1. Introduction

Antiseptics and disinfectants, whose worldwide consumption is increasing year by year, rank among the most intensively studied trace organic contaminants [1]. The wide range of their usage in daily urban activities, including dental hygiene, contributes to the continual release of these biologically active compounds into the environment in amounts that lack any control or restrictions. Quaternary ammonium compounds (QACs), such as hexadecylpyridinium chloride (HDP) and octenidine (OCT), phenolic derivatives (e.g., triclosan, TCS), and biguadines (e.g., chlorhexidine, CHX) belong among frequently used antiseptic compounds in toothpastes and mouthwashes and some of them were already detected in wastewater effluents [2,3].

The water solubility of these micropollutants has the potential to cause widespread contamination, usually at very low concentration levels (pg–µg/L). Due to the fact that wastewater treatment plant (WWTP) technologies are usually not designed for their removal, most disinfectants are not being eliminated [4,5]. Several studies demonstrated that antiseptics and disinfectants were released from WWTPs [2,6] and their ecotoxicological impact was observed. The increasing concentrations of disinfectants have caused changes in microbial communities in polluted rivers [7], sediments [8], as well as in activated sludge [9]. Microbial resistance and/or cross-resistance with antibiotics was already described for some disinfectants [4,10,11]. Michalíková et al. [12] revealed antiestrogenic and/or antiandrogenic properties of dental care antiseptics CHX, OCT, and also HDP. The fate of these compounds in the environment is not yet fully understood. For instance, the removal of CHX and HDP in WWTPs was studied by several authors [9,13,14,15,16] with the same general conclusion that these compounds are not biodegraded during wastewater treatment processes. The removal of OCT has not been investigated so far. Moreover, the stability under different physical and chemical conditions and resistance to hydrolysis has been presented as a benefit of this novel disinfectant [17].

Various anthropogenic pollutants persist in the environment and scientific interest is generally aimed at the impacts on organisms and human health and also mechanistic studies of their degradability. Besides physicochemical degradation, a lot of attention is focused on biodegradation studies. In particular, microorganisms possess a wide range of metabolic pathways that have been shown to be very effective in the decomposition of pollutants.

Ligninolytic (white-rot) fungi are excellent model degraders, well-known for their efficiency in the decomposition of a broad range of xenobiotics, even those resistant to bacterial breakdown or generally hardly biodegradable, e.g., polychlorinated biphenyls [18,19], polycyclic aromatic hydrocarbons [20,21], chlorobenzoic acids [22], explosives [23], and also the most toxic organic pollutant known so far—2,3,7,8-tetrachlorodibenzodioxin [24]. The degradation ability of ligninolytic fungi to decompose xenobiotics has also been documented for dyes, several endocrine disruptors, pharmaceuticals (including antibiotics), plasticizers, UV filters, etc. [25,26,27,28].

Ligninolytic fungi possess a unique extracellular enzyme apparatus with low substrate specificity naturally targeted toward the degradation of the aromatic moieties of lignin. These extracellular enzymes, such as lignin peroxidase, manganese-dependent peroxidase (MnP), and the phenol oxidase laccase (Lac), catalyze nonspecific one-electron radical oxidations and have been shown to transform a wide range of organic pollutants. TCS, a chlorinated antimicrobial compound, was shown to be degraded in vitro by ligninolytic enzymes [29,30]. Baborová et al. [31] demonstrated the transformation of polycyclic aromatic hydrocarbons by MnP from *Irpex lacteus* and proved the formation of oxidized metabolites and aromatic ring cleavage. Extracellular enzymes are assumed to be responsible for the main degradation ability of ligninolytic fungi. Nevertheless, several authors have proven the role of the intracellular cytochrome P-450 of ligninolytic fungi in the transformations, e.g., with the synthetic hormone 17*α*-ethynilestradiol [28] and chlorobenzoic acids [32].

The aim of this work was to investigate the biodegradability of CHX and OCT, which are used in oral care products and were identified in our previous research as endocrine disruptors, by two model ligninolytic fungal strains, *I. lacteus* and *Pleurotus ostreatus*, i.e., species with proven biodegradation capabilities. To the best of our knowledge, no biodegradation study of these disinfectants by ligninolytic fungi has ever been carried out. The biodegradability of CHX and OCT was studied in vivo with fungal cultures grown in a liquid medium, as well as in vitro with concentrated extracellular liquids rich in ligninolytic enzymes. The samples were evaluated for the presence of metabolites, which is rarely described in the literature.

## 2. Results and Discussion

### 2.1. In Vivo CHX and OCT Transformation

Both CHX and OCT are designed to act against microorganisms. Thus, the highest nontoxic concentrations of both analytes towards the fungal strains were investigated as the first step of the assessment. Each compound was added in dimethyl sulfoxide (DMSO, final concentration 0.5% of DMSO) to the medium with the fungal suspension, and the weight of the biomass was compared to a control culture (0.5% DMSO) after 7 and 14 days (data not shown). The final concentrations of 3 and 2 µg/mL of CHX and OCT, respectively, were established as nontoxic concentrations for the growth of the studied fungal cultures.

The relationship between the presence of extracellular enzymes and degradation rates is often monitored in order to explain a part of the complex in vivo degradation mechanisms [33]. Hence, the most abundant enzymes were monitored throughout the entire in vivo experiment to observe possible relationships. 

#### 2.1.1. Chlorhexidine

MnP was the major extracellular enzyme in the culture of *I. lacteus* and was produced continuously with the activity level of 5 ± 1 U/L during the whole 21-day experiment. Even though *I. lacteus* was found to also produce extracellular Lac [33], the enzyme activity measured in the culture medium was usually low [34,35], which corresponds with our findings. The dominant enzyme in the culture of *P. ostreatus* was Lac with the initial activity of 33 ± 4 U/L, which decreased about five times after three weeks of cultivation (see Table S1 for details).

**Figure 1:**
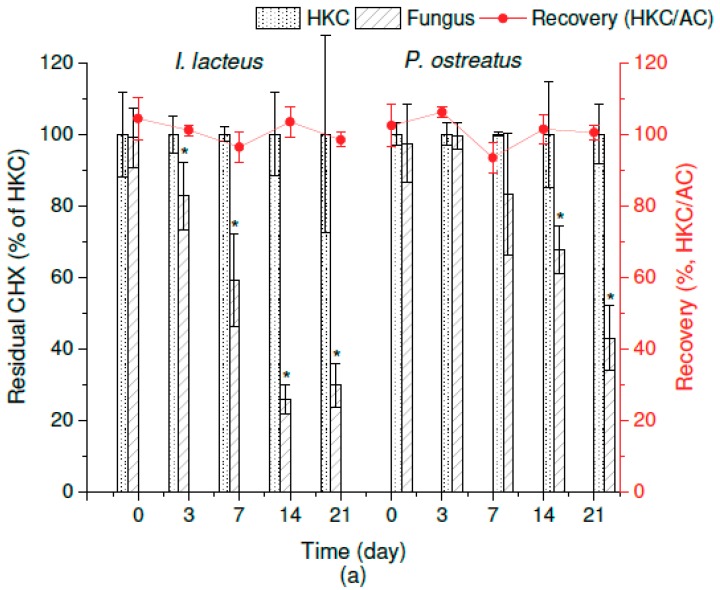

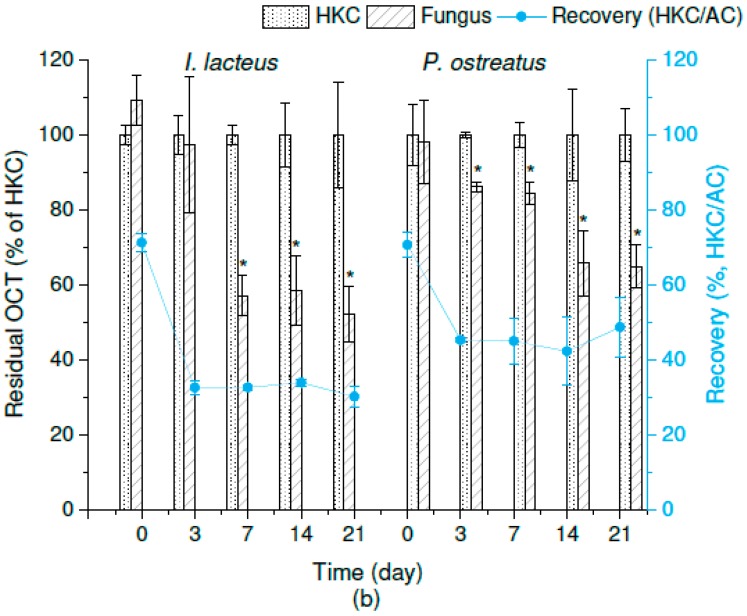
Residual concentration of (**a**) chlorhexidine (CHX) and (**b**) octenidine (OCT) after the 21-day in vivo degradation by *I. lacteus* and *P. ostreatus* related to the respective heat-killed controls (HKCs). The red (CHX) and blue (OCT) line graphs show extraction recovery during the experiment expressed as the HKC and abiotic control (AC) ratio. Error bars represent standard deviation (*n* = 3). The asterisk marks a significant difference between the individual harvesting day and the control group—0 d (post hoc Dunnett’s Test).

After 21 days of static in vivo cultivation of *I. lacteus* and *P. ostreatus* in a liquid medium, the residual amounts of CHX were 30% ± 6% and 43% ± 9%, respectively, in comparison with the respective heat-killed controls, HKCs (Figure 1a, left axis). The extraction recovery (the ratio of the concentration of the analyte determined in the HKC versus in the abiotic control − AC) indicated that 99% ± 2% and 100% ± 2% of CHX was extracted from the fungal cultures of *I. lacteus* and *P. ostreatus*, respectively (Figure 1a, right axis). 

Notably, the extraction solvent, 0.8% formic acid (FA) in 20% acetonitrile (ACN, *v*/*v*), together with sonication at elevated temperature improved CHX recovery from both fungal cultures by a factor of 1.4 (compared to the value obtained without any extraction). This result proposes the disruption of the ionic binding of the analyte to the negatively charged sites of the fungal cultures. The enhancement of the CHX recovery in acidic conditions are in accordance with the observation of Havlíková et al. [36].

A strong sorption phenomenon on the biomass of an activated sludge studied for its potential to remove CHX was observed under laboratory conditions [9]. The authors concluded that biosorption was mainly responsible for the CHX elimination. Similarly, a study of the mass balance of CHX in a WWTP revealed its 98% removal from the wastewater. In this specific case, the sorption to the sludge was the only mechanism of elimination and the authors highlighted the lack of CHX degradation [13]. The bioaccumulation of CHX in the lipids of both diatoms and bacteria in river biofilm communities was also observed and a subsequent stable isotope analysis indicated the absence of CHX mineralization [37,38]. Fortunato et al. [6] performed a degradation study with several adapted bacterial strains that were characterized and isolated from water samples in urban regions. CHX was found to be the most resistant and toxic compound in comparison with TCS and benzalkonium chloride. These observations emphasize the recalcitrant character of CHX and the low possibility of its removal from the environment. Microbial degradation of CHX was accomplished by bacterial isolates from an activated sludge in a study by Tanaka [39]. An 80% reduction of CHX antimicrobial activity was achieved and the structure where pyruvate is bound to the CHX molecule was proposed as a less active intermediate. 

#### 2.1.2. Octenidine

In the case of OCT, the residual concentration of the analyte reached values of 52% ± 7% and 65% ± 6% in comparison with the HKC after 21-day cultivation in the cultures of *I. lacteus* and *P. ostreatus*, respectively (Figure 1b, left axis). The activities of the enzymes were comparable with the values reached in the experiment with CHX. MnP activity (2.6 ± 0.8 U/L) remained nearly constant until the termination of the experiment with *I. lacteus,* while the initial activity of Lac of *P. ostreatus* (31.5 ± 0.5 U/L) was suppressed approximately 10 times after the 21-day degradation of OCT.

A comparison of the extraction yields of OCT in the AC and HKC revealed massive sorption of OCT on the biomass. The sorption of OCT was already evident at the beginning of the experiment (day 0)—it was possible to extract only about 70% of the analyte from the biomass, in both cultures (Figure 1b, right axis). At the end of the experiment, 70% ± 3% and 51% ± 8% of OCT was adsorbed in the cultures of *I. lacteus* and *P. ostreatus,* respectively. The analyte did not release from the fungal mycelium, even after the ultrasound-assisted extraction at elevated temperature in the acidified polar organic solution. Despite our best effort to achieve better extraction yields, it is not clear whether the extracellular enzymes of *I. lacteus* and *P. ostreatus* were responsible for the biodegradation of OCT in vivo or if the sorption was the only reason for its removal. Differences between HKC and live biomass (ANOVA, *p* < 0.05) favor the enzymatic transformation. However, the changes in the sorption capacity of the HKC samples caused by autoclaving might also play a role in the data interpretation. Unfortunately, information about the fate of OCT in the environment is insufficient so far. Nevertheless, the adsorption of different QAC on particulate matter is often discussed in the literature [2,14]. The characteristics of OCT, namely the positive charge in physiological conditions and the strong binding to the negatively charged sites of biological membranes [17,40], indicate that the interaction with the mycelium will be similar. 

### 2.2. Extracellular In Vitro Transformation 

Detectable activities of the extracellular enzymes MnP and Lac were recorded during the in vivo transformation experiments with *I. lacteus* and *P. ostreatus*, respectively (see Table S2 for details). Transformation associated with the activities of MnP and Lac was further investigated employing a concentrated extracellular liquid of the eight-day-old malt extract-glucose (MEG) culture of *I. lacteus* supplemented with Mn^2+^ and a hydrogen peroxide-generating system and the eight-day-old MEG culture of *P. ostreatus,* respectively. The initial activities of MnP and Lac in the reaction mixture were 60 U/L and 120 U/L, respectively, and they did not decrease below 25% of their initial value during the whole 192-h experiment. 

#### 2.2.1. Chlorhexidine

**Figure 2:**
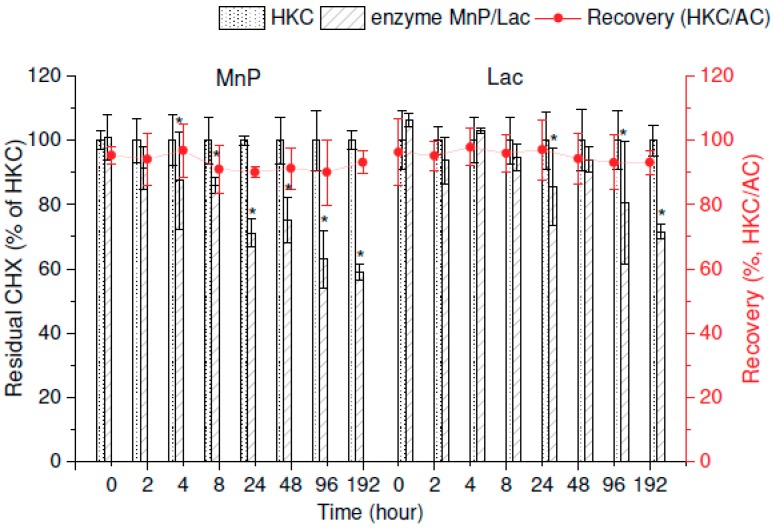
In vitro degradation of chlorhexidine (CHX) in concentrated extracellular liquids of *I. lacteus* (manganese-dependent peroxidase, MnP) and *P. ostreatus* (laccase, Lac). Initial concentration of CHX was 5 µg/mL in both experiments. The red line graphs show the recovery of CHX extraction during the experiment expressed as the heat-killed control (HKC) and abiotic control (AC) ratio. Error bars represent standard deviation (*n* = 3). The asterisk marks a significant difference between the individual harvesting day and the control group—0 d (post-hoc Dunnett’s Test).

The residual amounts of CHX related to the HKC after in vitro incubation with MnP and Lac were 59% ± 2% and 72% ± 2%, respectively (Figure 2, left axis). Significant removal of CHX was recorded after 4 h in the case of MnP and after 24 h in samples enriched by Lac (ANOVA, *p* < 0.05). The percentage of the extraction recovery reached 94% ± 3% on average (Figure 2, right axis). 

Several studies have documented that the degradation of various recalcitrant pollutants by ligninolytic enzymes is usually very fast (in the range of hours) and mostly up to 100% effective [20,41,42]. Our results show that MnP and Lac were able to catalyze only 41% ± 2% and 28% ± 2% of the transformation of CHX after 192 h, respectively. Interestingly, even though the activities of the enzymes were higher than in the case of the in vivo experiment, the degradation achieved with the whole fungal culture was faster. The presence of enzymes bound to the mycelia (e.g., Lac), which were not harvested for the in vitro enzyme experiment and thus were available only in the in vivo incubation (where they were unaccounted for by the enzyme activity assays), could be a possible explanation [28]. In conclusion, the in vitro results suggest both mechanisms—sorption and biotransformation—might be involved in the removal of CHX.

#### 2.2.2. Octenidine

**Figure 3:**
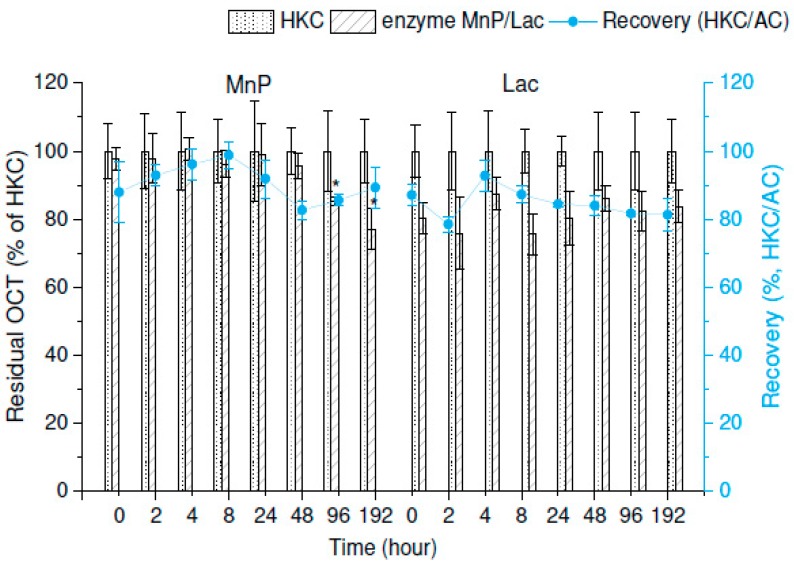
In vitro degradation of octenidine (OCT) in concentrated extracellular liquids of *I. lacteus* (manganese-dependent peroxidase, MnP) and *P. ostreatus* (laccase, Lac). Initial concentration of OCT was 5 µg/mL in both experiments. The blue line graphs show the recovery of OCT extraction during the experiment expressed as the heat-killed control (HKC) and abiotic control (AC) ratio. Error bars represent standard deviation (*n* = 3). The asterisk marks a significant difference between the individual harvesting day and the control group—0 d (post-hoc Dunnett’s Test).

The removal of OCT was not recorded until the 96th hour of incubation in the case of MnP (ANOVA, *p* < 0.05). No decrease of the initial amount of OCT in time was observed with samples containing Lac after 192 h, ANOVA, *p* > 0.05 (see Figure 3 for details). Due to the sorption of the analyte observable for the HKC and the high variability of the data, the participation of MnP and Lac in OCT biodegradation is disputable. It is also important to note that the crude extracellular liquid is a complex matrix containing concentrated proteins and the involvement of other extracellular enzymes not considered in the activity assays might be another possible explanation for the removal of OCT. We argue that the slight decrease in the residual concentration relates rather to the sorption (~20%). This experiment did not support the theory that extracellular enzymes are responsible for the removal of OCT, but points toward the adsorption mechanism.

Total removal was achieved for neither CHX nor OCT under any conditions and the biotransformation of CHX in vitro was considered slow and less efficient in comparison with previous studies dealing with the biodegradation of recalcitrant pollutants by white-rot fungi. For instance, *P. ostreatus* was found to decompose nearly 100% of a mixture of polychlorinated biphenyls in MEG and low-nitrogen mineral medium after 42 days of incubation [18]. An excellent degradation rate (≥88%) of endocrine-disrupting compounds as well as the suppression of estrogenic activity were accomplished with various ligninolytic fungal strains in 14 days [26]. In addition, Muzikář et al. [22] demonstrated that selected ligninolytic fungal strains are powerful degraders of chlorobenzoic acids under both model liquid conditions and in contaminated soil—the chlorobenzoic acids reached 85%–99% degradation within 60 days.

### 2.3. Identification of Metabolites

**Figure 4:**
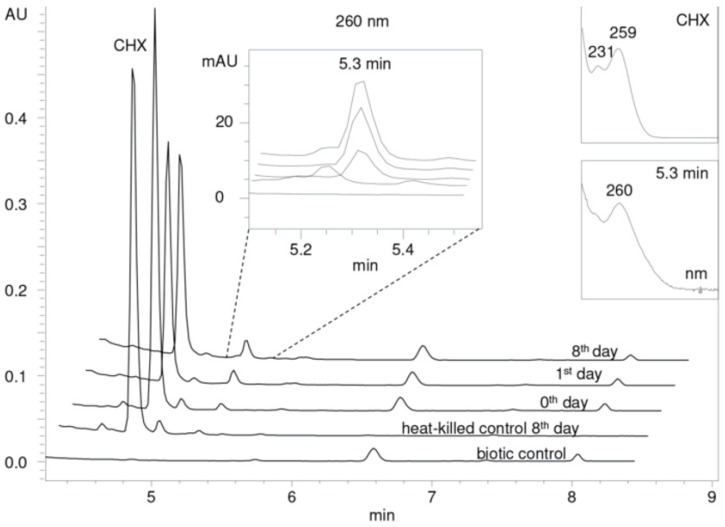
LC-UV plots of degradation samples acquired during in vitro chlorhexidine (CHX) degradation in the concentrated extracellular liquid of *I. lacteus* supplemented with Mn^2+^ and H_2_O_2_. The UV absorption spectra of specific peaks are given in the insets.

**Figure 5:**
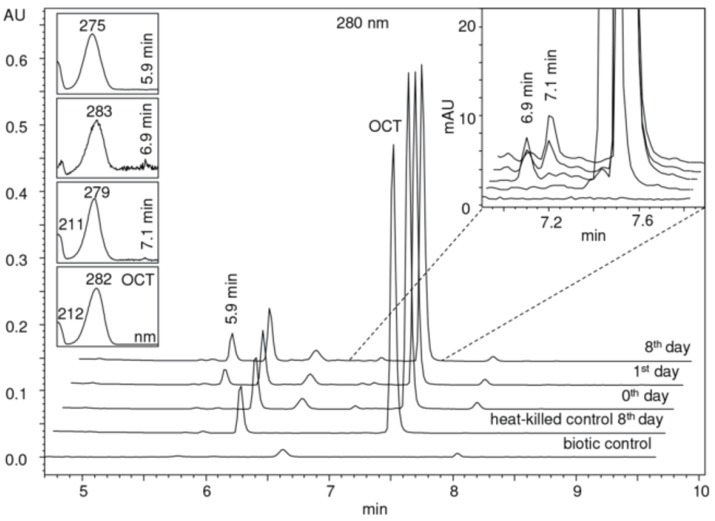
LC-UV plots of degradation samples acquired during in vitro octenidine (OCT) degradation in the concentrated extracellular liquid of *I. lacteus* supplemented with Mn^2+^ and H_2_O_2_. The UV absorption spectra of specific peaks are given in the insets.

In vivo experiments did not reveal any transformation products. Detectable amounts of metabolites were recorded only during the in vitro tests carried out with both enzymes. Figure 4 and Figure 5 show the LC-UV chromatograms obtained during the 8-day experiment spiked with 50 µg/mL of CHX and OCT, respectively (for the sake of clarity only the experiment with one of the enzymes—MnP—is displayed because the same products were observed). All peaks, especially those that did not appear in the control samples, were carefully assessed by nontargeted LC-MS analysis.

#### 2.3.1. Chlorhexidine

An additional peak with the retention time (R_t_) 5.3 min, whose area increased within the degradation experiment, was recorded in the chromatogram (Figure 4). A similar profile of the UV absorption spectra (λ_max_ = 260 nm) suggested a relationship with CHX (R_t_ = 4.8 min).

LC-MS analysis (full-scan mode) revealed the major *m*/*z* 258.2 [M + 2H]^2+^ and 515.2 [M + H]^+^ for the peak with the R_t_ = 5.3 min. Detailed characteristics of the ESI^+^ mass spectra of CHX and its transformation product, the comparison of their *m*/*z* values with the theoretical ones, as well as the suggested structures of the metabolites, are given in Table 1. The specific isotopic pattern of the CHX metabolite (ions 258.2; 259.0; 260.0 and 515.2; 517.1; 519.1) indicated the existence of multiple chlorine atoms in the molecule. According to the presented structure (Table 1, R_t_ 5.3 min), oxidation and simultaneous dehydrogenation seemed to be involved in the CHX transformation. Hydroxylation is a common reaction facilitated by ligninolytic enzymes possessing low substrate specificity [43]. Dehydrogenation was described for the degradation of steroid compounds by *P. ostreatus* [28]. Our observation suggests multiple dehydrogenation of the alkyl chain of CHX. Product ion spectra of [M + 2H]^2+^ and [M + H]^+^ obtained by fragmentation in a linear ion trap and the description of the main fragments are proposed in Figure S1.

Several authors tracked CHX degradation by bacterial isolates *(Pseudomonas* sp.) from activated sludge [15,16,44]. As a result, Tanaka et al. [39] proposed two ways of CHX transformation. The first is a direct degradation to *p*-chlorophenylurea (PCPU) and *p*-chloroaniline (PCA). The second pathway assumes an intermediate in which pyruvate is bound to the CHX molecule (*m*/*z* 531 [M + H]^+^). PCA and PCPU are also known products of the hydrolysis of CHX under acidic or alkaline conditions [45,46]. We did not observe any detectable amount of PCA or PCPU in the degradation samples.

#### 2.3.2. Octenidine

Three additional peaks appeared at R_t_ 5.9, 6.9, and 7.1 min (Figure 5). The detailed mass spectral information of all observed OCT transformation products together with the comparison of the proposed chemical structures of OCT metabolites and their theoretical *m*/*z* values are summarized in Table 1. The product assigned with the R_t_ = 5.9 min, distinguished by LC-MS as the *m*/*z* 220.2 [M + 2H]^2+^, corresponds to the loss of the octyl moiety from the OCT structure. The amount of this transformation product clearly increased within incubation time (Figure 5). Notably, detectable amounts were also recorded in the HKC and AC samples but their abundancies remained constant. This phenomenon can be explained as a physicochemical degradation of OCT that might be accelerated in the presence of ligninolytic enzymes. The peaks with the R_t_ = 6.9 min and R_t_ = 7.1 min occurred only in trace amounts and their baseline resolution was not achieved. Interestingly, one of these metabolites (R_t_ = 6.9 min) started to disappear after several hours, while the amount of the second compound (R_t_ = 7.1 min) increased after 24 h of incubation, indicating a less stable intermediate. A hydroxylated and hydroxylated/dehydrogenated structure was proposed for major ions 284.2 [M + 2H]^2+^ and 283.2 [M + 2H]^2+^ assessed by LC-MS, respectively (see Table 1 for details). Product ion spectra of all the transformation products and the description of their fragmentation products are displayed in Figures S2–S5.

The specific example of QAC degradation by ligninolytic fungi is lacking in the literature. On the other hand, the potential of the monooxygenases and dioxygenases of *Rhodobacter* spp. to biotransform HDP, a structurally similar compound, via dealkylation and/or benzene ring oxidation was hypothesized in the study of Nguyen et. al. [14]. Three main mechanisms of hydroxylation were described for the biotransformation of QACs in aerobic conditions [4], but the fate of these compounds under anaerobic conditions (e.g., aquatic sediments where they are usually adsorbed) remains unknown.

## 3. Materials and Methods

### 3.1. Chemicals

CHX (99.5%) was obtained from Merck (Darmstadt, Germany). OCT dihydrochloride (98%) was obtained from Alfa Aesar (Ward Hill, MA, USA). The tested compounds were diluted in DMSO (99.9%, Merck). Malt extract broth (Oxoid, Basingstoke, UK) and glucose (Penta, Prague, Czech Republic) were used for the MEG medium for fungal cultivation. ACN (HPLC grade and LC-MS grade, VWR, Prague, Czech Republic), milli-Q water prepared by the Direct-Q^®^ water purification system (18.2 MΩ·cm, Merck), FA (98%, Penta, Czech Republic), FA (LC-MS grade, Honeywell, Charlotte, NC, USA), and trifluoroacetic acid (TFA ≥ 99%, Merck) were used for quantitative analyses.

### 3.2. Cultivation of Organisms and Degradation Tests

#### 3.2.1. Fungal Cultivation

Ligninolytic fungal strains *P. ostreatus* 3004 CCBAS 278 and *I. lacteus* 617/93 were obtained from the Culture Collection of Basidiomycetes of the Czech Academy of Sciences (Prague, Czech Republic). One week-grown fungi were maintained on MEG (0.5% malt extract broth, 1% glucose) agar plate and stored at 4 °C. One week before the degradation tests, fungal inocula containing 3 mycelial plugs (0.7 mm Ø) of *I. lacteus* and *P. ostreatus* were grown in 20 mL of the MEG medium in 250 mL Erlenmeyer flasks (ErF) under static conditions for 5 and 7 days, respectively. The inocula were homogenized by Ultra-Turrax T 25 (IKA, Staufen, Germany).

#### 3.2.2. In Vivo Transformation

Aliquots (1 mL) of the homogenized fungal suspension from Section 3.2.1. were used to inoculate static cultures (20 mL of the MEG medium, 250 mL ErF). In vivo cultures were contaminated with the highest nontoxic concentration of CHX (3 µg/mL in 0.5% DMSO) or OCT (2 µg/mL in 0.5% DMSO) three days after inoculation, all in triplicate. Biotic controls (BC, fungal culture with 0.5% DMSO) and HKCs were prepared in parallel. HKCs were grown for the same amount of time as the respective fungal cultures and subsequently inactivated by sterilization (121 °C, 30 min). After that, they were contaminated and incubated for the same amount of time as the active cultures. The samples and corresponding controls were harvested after 0, 3, 7, 14, and 21 days of cultivation (static conditions, at 28 °C in the dark) and then a quantitative analysis was performed (see Section 3.4.1).

#### 3.2.3. In Vitro Transformation with Concentrated Extracellular Liquids

Extracellular enzymes produced by the studied fungal strains were used for in vitro transformation tests. *I. lacteus* and *P. ostreatus* were grown in the MEG medium (20 mL in 250 mL ErF) for 8 days. The cultures were filtered through a 0.22 µm cellulose membrane (Whatman, Little Chalfont, UK). The targeted enzymes were concentrated 100-fold using 10 kDa cut-off membrane (Whatman) at 4 °C. The crude concentrated extracellular liquids were used for in vitro transformation experiments. The experiment with MnP from the *I. lacteus* strain was performed in 2 mL reaction mixtures containing MnP at initial activity of 60 U/L, 50 mM malonate buffer (pH 4.5), 1 mM MnSO_4_, H_2_O_2_-generating system (30 mM glucose, 60 U/L of glucose oxidase), and 5 µg/mL of CHX or OCT, respectively. In the case of the experiment with Lac from the *P. ostreatus* strain, the reaction mixtures contained Lac with initial activity of 120 U/L, 60 mM acetate buffer (pH 5.0), and 5 µg/mL of either CHX or OCT. All samples were prepared in triplicate. ACs were prepared with distilled water instead of the enzyme. The enzyme was inactivated (100 °C, 30 min) to prepare corresponding HKCs; BCs contained 5% DMSO instead of the analytes. The samples and the respective controls were incubated for 0, 2, 4, 8, 24, 48, 96, and 192 h at 28 °C in the dark, shaken at 80 rpm on a rotary shaker. All samples were then extracted and the residual concentration of the analytes was determined (Section 3.4.1).

### 3.3. Enzyme Activities

Extracellular ligninolytic enzymes were measured during the cultivation, enzyme preparations for the in vitro experiments, and all degradation experiments. Lac was determined by the 2,2′-azino-di-[3-ethylbenzthiazoline sulfonate (6)] (Merck) oxidation test [47]. MnP and manganese-independent peroxidase were assessed with 2,6-dimethoxyphenol (Merck) as the substrate [48]. One enzyme unit produced 1 µmol of the reaction product per minute under the reaction conditions.

### 3.4. Chemical Analyses

#### 3.4.1. Quantitative Analyses 

In vivo transformation samples as well as the controls were homogenized by the Ultra-Turrax T 25 and the fungal suspension was sonicated with 20 mL of an extraction solution containing 0.8% FA in 20% ACN (*v*/*v*) at 70 °C for 45 min. The extracted suspension was centrifuged (4136× *g*, 15 min) and the supernatant was measured by LC-UV.

In vitro transformation samples were added with 2 mL of the extraction solution and sonicated at 70 °C for 45 min. The extracts were centrifuged (6000× *g*, 10 min) and the supernatant was analyzed by LC-UV.

The Waters Alliance 2695 LC system (Waters, Milford, MA, USA) equipped with a diode-array detector (Waters 2996) was used for the analysis. The analytes were separated on the XBridge C18 (4.6 × 150 mm, 3.5 μm) column (Waters). The mobile phase consisted of 100% ACN (A) and 10% ACN with 0.1% TFA (*v*/*v*) (B), the flow rate was 0.8 mL/min, and gradient elution was applied (min/% of A): 0/20, 8–10/95, 15/20. Column temperature was set at 35 °C, sample injection was 10 µL. Detection wavelengths for CHX and OCT were 260 nm and 280 nm, respectively.

#### 3.4.2. Identification of Metabolites

The metabolites in all transformation samples were investigated by LC-MS analysis using the NexeraXR ultra-high performance liquid chromatograph (Shimadzu, Kyoto, Japan) coupled via electrospray ionization (ESI) to the QTrap 4500 mass spectrometer (Sciex, Framingham, MA, USA). The Analyst 1.6.3. software was used for data evaluation. Separation was achieved on the Cortecs T3 C18 column (150 mm × 3 mm, 2.7 µm) at 0.4 mL/min using a mobile phase composed of 0.1% FA in water (A) and ACN (B). The linear gradient was as follows (min/% of A): 0/10, 8/70, 9–12/100, 12–15/20. The mass spectrometer operated in the positive mode. Curtain gas, ion spray voltage, vaporizer temperature, ion source gas 1, and ion source gas 2 were set at 30 psi, 5.5 kV, 450 °C, 40 psi, and 50 psi, respectively. Full scan analysis in the mass range of 100–600 *m*/*z* was used for metabolite identification. Enhanced product ion, tandem mass spectrometry, and enhanced resolution scans with alternated collision energies and mass ranges were used to study the metabolites in more detail.

### 3.5. Statistical Analysis

The one-way analyses of variance (ANOVA) and subsequent Dunnett’s tests were run to identify the pairs with significant differences. The differences were considered significant at *p* < 0.05. Microsoft excel 2016 (Redmond, WA, USA) was used for data handling.

## 4. Conclusions

Our results indicate that the metabolic transformation of CHX might be associated with the activity of extracellular enzymes of ligninolytic fungi, MnP and Lac, but only slow removal of the pollutant was observed under the model conditions. Moreover, the mechanism elucidating the metabolism of OCT remains unexplained since the majority of the analyte was adsorbed to the mycelial matter. For the first time, metabolites indicating enzymatic transformation of both CHX and OCT were detected and their chemical structures were proposed. However, complete biodegradation by the ligninolytic fungi was not achieved for any of the studied analytes, which emphasizes their recalcitrant character. Taken together with the substantial increase in the production and their worldwide consumption during everyday household activities, there is little prospect of CHX and OCT being removed from the environment. Activated sludge and agricultural lands are expected to be the environments impacted by these compounds in real circumstances. The conclusions of this study highlight that biodegradation studies of newly developed and extensively consumed synthetic compounds are crucial to predict their fate in the environment.

## Figures and Tables

**Table 1:** Characterization of the detected chlorhexidine (CHX) and octenidine (OCT) metabolites by nontargeted LC-MS.

R_t_ [min]	Suggested Structure	Theoretical Mass (Monois.) *m*/*z*	Mass Spectra Characteristics (ESI+) *m*/*z* (Intensity, %)
CHX 4.8	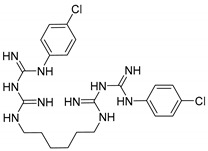	505.2105 253.1089	253.2 (100); 254.1 (98); 255.1 (29); 505.2 (31); 507.1 (22); 509.1 (5); 336.1 (20); 338.2 (7); 319 (14)
5.3	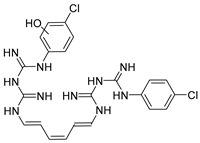	515.1584 258.0829	258.2 (100); 259.0 (70); 260.0 (13); 515.2 (59.7); 517.1 (44); 519.1 (9.7); 346.0 (6.2); 348.1 (4.9); 498.0 (5.4); 500.1 (3.7); 502.1 (1)
OCT 7.5	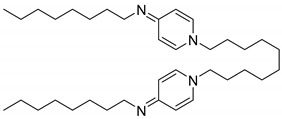	551.5047 275.7524	276.3 (100); 551.4 (17.6); 345.4 (1.6)
5.9	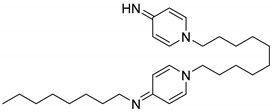	439.3795 220.1934	220.2 (100); 439.4 (13.7); 345.3 (3.1)
6.9	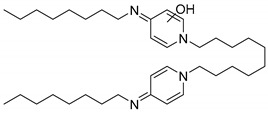	567.4996 284.2534	284.2 (100); 230.3 (459) (46.7); 488.2 (4.2); 459.5 (2.7); 567.3 (2.1); 276.2 (1)
7.1	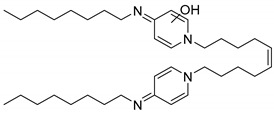	565.4840 283.2455	283.2 (100); 565.3 (5.4); 275.3 (4.6); 269.3 (3.9); 220.7 (1.5)

## Supplementary Materials

The following are available online, Table S1: Activity of manganese-dependent peroxidase (MnP) from *I. lacteus* and laccase (Lac) from *P. ostreatus* during in vivo degradation of octenidine (OCT) and chlorhexidine (CHX); Table S2: Activity of manganese-dependent peroxidase (MnP) from *I. lacteus* and laccase (Lac) from *P. ostreatus* during in vitro degradation of octenidine (OCT) and chlorhexidine (CHX); Figure S1: Product ion spectra and suggested fragments of (a) *m*/*z* 515.2 [M + H]^+^ and (b) *m*/*z* 258.2 [M + 2H]^2+^; Figure S2: (a) mass spectrum of the peak with Rt = 5.9 min, *m*/*z* 439.4 [M + H]^+^ (b) product ion spectra and suggested fragments of *m*/*z* 439.4 [M + H] ^+^; Figure S3: Product ion spectra and suggested fragments of (a) *m*/*z* 567.5 [M + H]^+^ and (b) *m*/*z* 284.3 [M + 2H]^2+^; Figure S4: (a) mass spectrum of the peak with R_t_ = 7.1 min, *m*/*z* 283.2 [M + H]^+^ (b) product ion spectra and suggested fragments of *m/z* 565.5 [M + H]^+^; Figure S5: Product ion spectra and suggested fragments of (a) *m*/*z* 565.5 [M + H]^+^ and (b) *m*/*z* 283.3 [M + 2H]^2+^.
