# Supplementary material for: Biodegradability of Dental Care Antimicrobial Agents Chlorhexidine and Octenidine by Ligninolytic Fungi
Source: Molecules. 2020 Jan 18;25(2):400. doi: 10.3390/molecules25020400 (PMC7024351; doi:10.3390/molecules25020400)
Supplement: Supplementary file 1 [file molecules-25-00400-s001.pdf]

Supplementary Data

Biodegradation of dental care antimicrobial agents chlorhexidine and octenidine by ligninolytic fungi

Lucie Linhartová [1,2], Klára Michalíková [1,2], Kamila Šrédlová [1,2] and Tomáš Cajthaml [1,2,*]

[1] Laboratory of environmental biotechnology, Institute of Microbiology of the Czech Academy of Sciences, Vídeňská 1083, CZ-14220 Prague 4, Czech Republic; lucie.linhartova@biomed.cas.cz (L.L.); klara.michalikova@biomed.cas.cz (K.M.); kamila.sredlova@biomed.cas.cz (K.Š.)

[2] Faculty of Science, Institute for Environmental Studies, Charles University, Benátská 2, CZ-12801 Prague 2, Czech Republic

* Correspondence: cajthaml@biomed.cas.cz

The list of supporting information:

Table S1: Activity of manganese-dependent peroxidase (MnP) from *I. lacteus* and laccase (Lac) from *P. ostreatus* during *in vivo* degradation of octenidine (OCT) and chlorhexidine (CHX).
Table S2: Activity of manganese-dependent peroxidase (MnP) from *I. lacteus* and laccase (Lac) from *P. ostreatus* during *in vitro* degradation of octenidine (OCT) and chlorhexidine (CHX).
Figure S1: Product ion spectra and suggested fragments of (a) *m/z* 515.2 $[M+H]^+$ and (b) *m/z* 258.2 $[M+2H]^{2+}$.
Figure S2: (a) mass spectrum of the peak with R_t = 5.9 min, m/z 439.4 $[M+H]^+$ (b) product ion spectra and suggested fragments of m/z 439.4 $[M+H]^+$.
Figure S3: Product ion spectra and suggested fragments of (a) *m/z* 567.5 $[M+H]^+$ and (b) *m/z* 284.3 $[M+2H]^{2+}$.
Figure S4: (a) mass spectrum of the peak with R_t = 7.1 min, *m/z* 283.2 $[M+H]^+$ (b) product ion spectra and suggested fragments of *m/z* 565.5 $[M+H]^+$.
Figure S5: Product ion spectra and suggested fragments of (a) *m/z* 565.5 $[M+H]^+$ and (b) *m/z* 283.3 $[M+2H]^{2+}$.

Table S1. Activity of manganese-dependent peroxidase (MnP) from *I. lacteus* and laccase (Lac) from *P. ostreatus* during *in vivo* degradation of octenidine (OCT) and chlorhexidine (CHX).

**Degradation**	**OCT**		**CHX**	
**Time**	**MnP (U/l)**	**Lac (U/l)**	**MnP (U/l)**	**Lac (U/l)**
0 d	3.6 ± 0.4	31.5 ± 0.5	4.6 ± 0.5	33 ± 4
3 d	3.0 ± 0.4	17.2 ± 0.5	6.2 ± 0.5	15 ± 3
7 d	1.5 ± 0.5	14.1 ± 0.1	5.7 ± 0.3	14.1 ± 0.7
14 d	2.5 ± 0.9	10.8 ± 0.1	6.3 ± 0.5	9.1 ± 0.4
21 d	2.5 ± 0.5	1.8 ± 0	4 ± 1	5.1 ± 0.1

Data are means ± SD (n=3).

Table S2. Activity of manganese-dependent peroxidase (MnP) from *I. lacteus* and laccase (Lac) from *P. ostreatus* during *in vitro* degradation of octenidine (OCT) and chlorhexidine (CHX).

**Degradation**	**OCT**		**CHX**	
**Time**	**MnP (U/l)**	**Lac (U/l)**	**MnP (U/l)**	**Lac (U/l)**
0 h	60 ± 1	120 ± 2	60 ± 2	120 ± 7
2 h	58 ± 2	119 ± 3	59 ± 4	118 ± 5
4 h	57 ± 2	118 ± 8	57 ± 5	116 ± 5
8 h	58 ± 2	105 ± 12	57 ± 3	110 ± 8
24 h	58 ± 8	79 ± 1	56 ± 5	86 ± 7
48 h	52 ± 4	55 ± 2	54 ± 3	62 ± 3
96 h	30 ± 5	47 ± 1	32 ± 7	42 ± 5
192 h	17 ± 2	33 ± 0	21 ± 4	31 ± 4

Data are means ± SD (n=3).

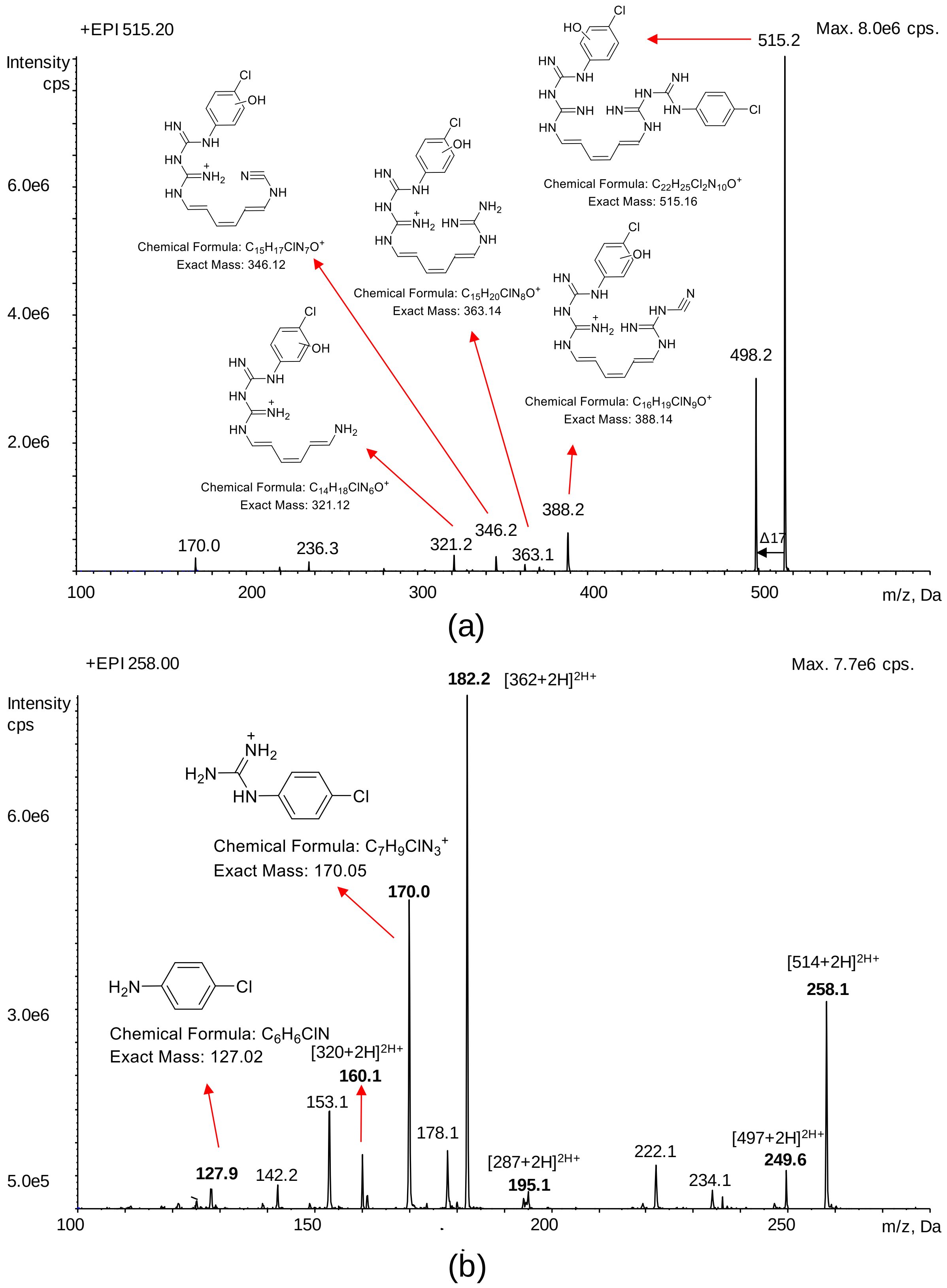

Figure S1: Product ion spectra and suggested fragments of (a) *m/z* 515.2 $[M+H]^+$ and (b) *m/z* 258.2 $[M+2H]^{2+}$.

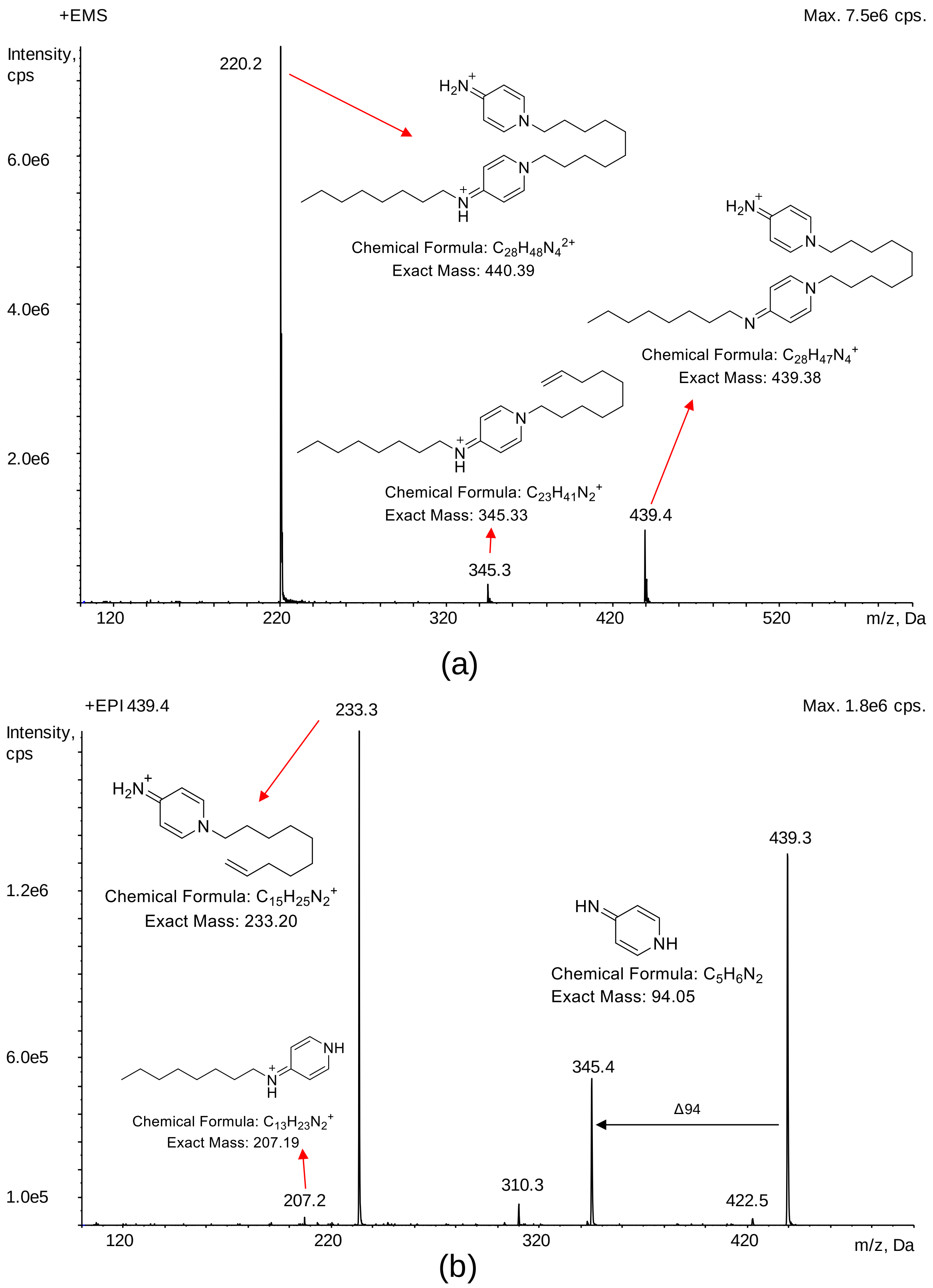

Figure S2: (a) mass spectrum of the peak with R_t = 5.9 min, *m/z* 439.4 $[M+H]^+$ (b) product ion spectra and suggested fragments of *m/z* 439.4 $[M+H]^+$.

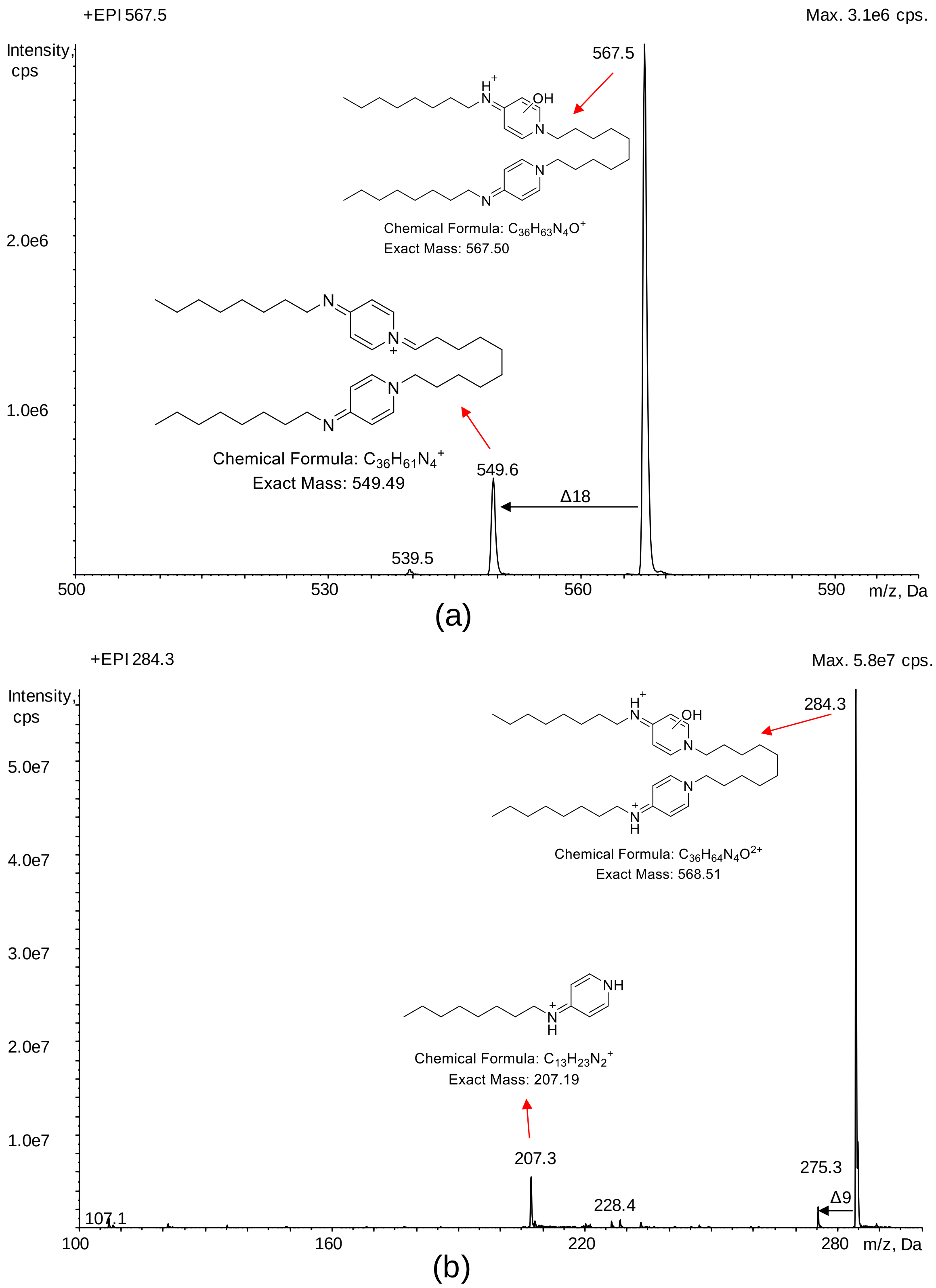

Figure S3: Product ion spectra and suggested fragments of (a) *m/z* 567.5 $[M+H]^+$ and (b) *m/z* 284.3 $[M+2H]^{2+}$.

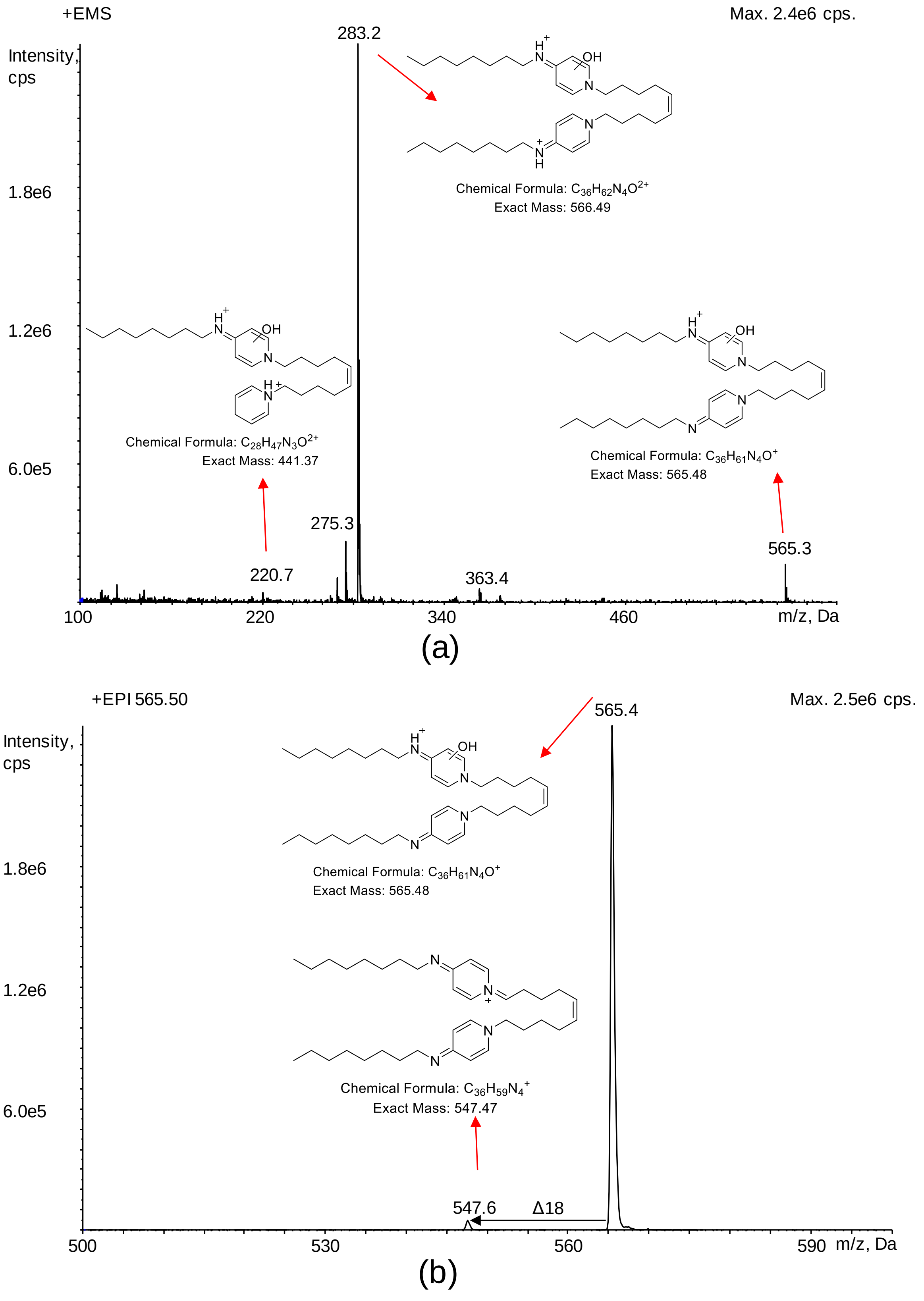

Figure S4: (a) mass spectrum of the peak with R_t = 7.1 min, *m*/*z* 283.2 $[M+H]^+$ (b) product ion spectra and suggested fragments of *m*/*z* 565.5 $[M+H]^+$.

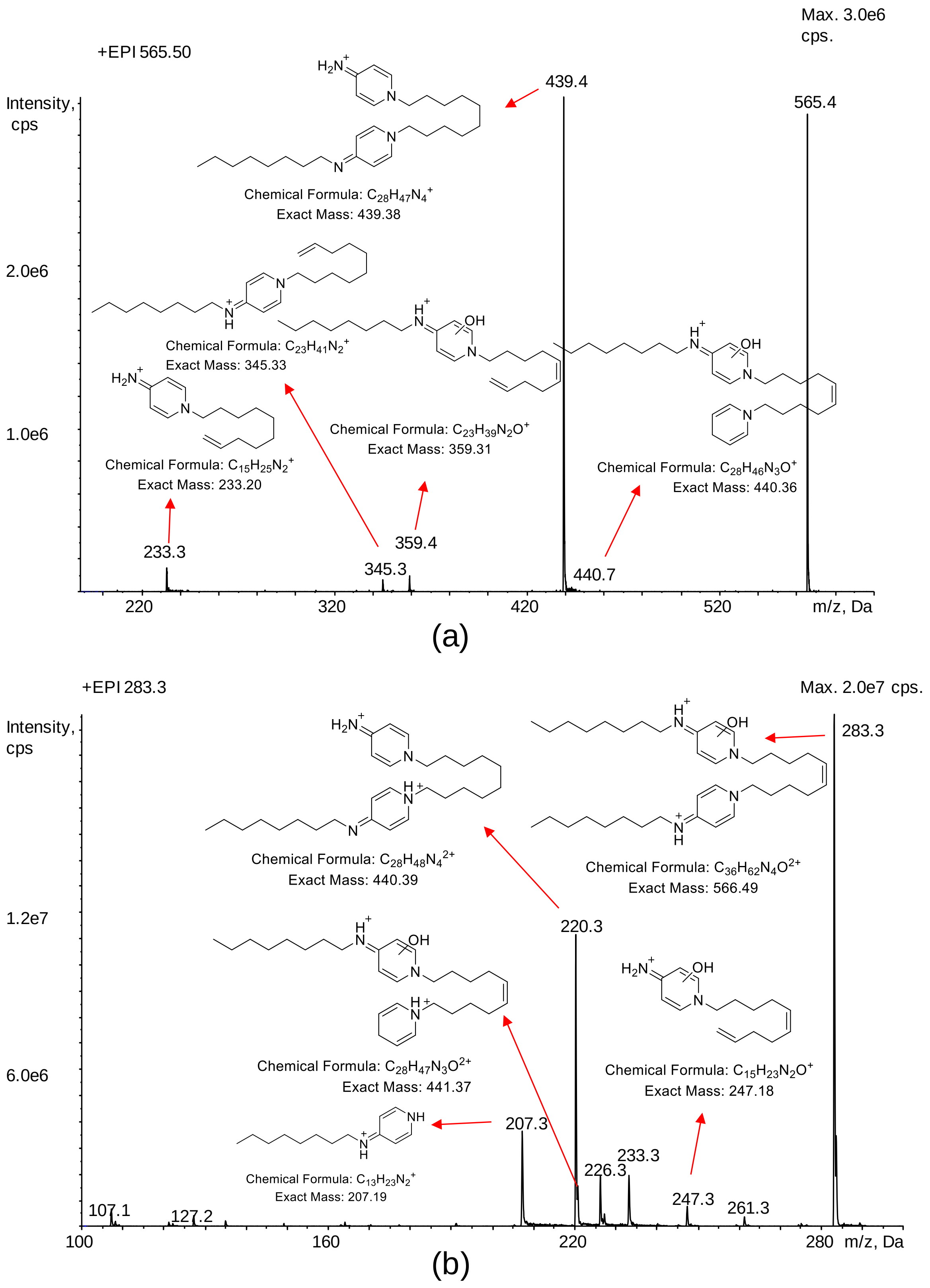

Figure S5: Product ion spectra and suggested fragments of (a) *m/z* 565.5 $[M+H]^+$ and (b) *m/z* 283.3 $[M+2H]^{2+}$.
